# Integrating Mutation-Derived and Expression Features from Single-Cell RNA Sequencing: Pitfalls of Standard Cross-Validation in Small-Cohort Settings

**DOI:** 10.3390/ijms27146429

**Published:** 2026-07-20

**Authors:** Aidyn Kunikeyev, Amankeldi A. Salybekov, Aigerim Yerimbetova, Batyrkhan Omarov

**Affiliations:** 1 Institute of Automation and Information Technologies, Satbayev University, Almaty 050013, Kazakhstan; 2 Regenerative Medicine Division, Cell and Gene Therapy Department, Qazaq Institute of Innovative Medicine, Astana 010000, Kazakhstan; amansaab0@gmail.com; 3 Institute of Information and Computational Technologies, Science Committee of the Ministry of Science and Higher Education of the Republic of Kazakhstan (CS MSHE RK), Almaty 050010, Kazakhstan; 4 School of Engineering and Information Technology, META University, Almaty 050012, Kazakhstan; 5 Department of Mathematical and Computer Modeling, International Information Technology University, Almaty 050040, Kazakhstan; b.omarov@iitu.edu.kz

**Keywords:** single-cell RNA sequencing, RNA-seq variant calling, GATK, mutation-derived gene-burden features, gene expression, leakage-safe validation, group-aware cross-validation, machine learning, expression–mutation integration, wound healing

## Abstract

Single-cell RNA sequencing (scRNA-seq) studies increasingly combine expression-based and mutation-derived signals, but small-cohort designs with repeated runs from the same biological unit can make standard cross-validation overly optimistic. We reanalyzed PRJNA736095 (14 SRR runs from 7 GSM/donor proxies) using a GATK-centered RNA-seq variant-calling and gene-burden workflow, then evaluated mutation-derived, expression-only, and combined feature sets with leakage-safe preprocessing inside each validation fold. Run-level repeated stratified cross-validation showed high within-dataset separability for GATK gene-burden features (balanced accuracy 0.973 +/− 0.113), but GSM-grouped leave-one-GSM-out validation reduced balanced accuracy to 0.708 and exact GSM-level permutation testing was not significant (*p* = 0.257). Expression-only and combined feature sets did not improve GSM-grouped balanced accuracy over the variant-only branch. Expression–mutation marker overlap was not significant after FDR correction, and public external datasets were used only to define feasibility or processed biological context rather than as strong external classifier validation. These findings position the workflow as an auditable, hypothesis-generating framework and highlight pitfalls of standard cross-validation in small-cohort scRNA-seq machine-learning analyses.

## 1. Introduction

Single-cell RNA sequencing (scRNA-seq) has transformed the study of cellular heterogeneity by enabling high-resolution profiling of transcriptional states across complex biological systems. Recent single-cell atlases have provided detailed insights into tissue organization, disease progression, regenerative processes, wound healing, and immune response dynamics [1,2,3]. In parallel, machine learning (ML) techniques have become essential tools for high-dimensional genomic data analysis, including classification, exploratory marker prioritization, and feature selection [4,5].

Despite these advances, most computational approaches in scRNA-seq analysis focus primarily on gene expression profiles, while mutation-derived signals remain underexplored. Recent studies have demonstrated that variant calling from scRNA-seq data can provide additional information about cellular identity and genetic heterogeneity [6,7]. However, in the majority of existing works, gene expression and mutation-derived features are analyzed independently, without explicitly modeling their relationships. This limitation restricts the ability to capture complementary biological signals across different molecular layers.

The integration of heterogeneous biological data sources, commonly referred to as multi-omics integration, has emerged as a key direction in biomedical data science [8,9]. Existing approaches include statistical integration frameworks, deep learning models, and graph-based methods. However, most of these approaches are designed for bulk sequencing data or large cohorts, where sufficient sample size mitigates overfitting risks. Their direct application to small-cohort scRNA-seq datasets remains problematic due to limited sample size and high feature dimensionality.

A critical challenge in biomedical machine learning is data leakage, which can lead to substantial overestimation of model performance. In scRNA-seq datasets, multiple samples or runs may originate from the same donor or metadata group. Standard cross-validation can then split related observations across training and test sets, introducing hidden dependencies that inflate apparent predictive performance on the dataset [10,11,12]. Recent studies emphasize structured validation strategies and reproducibility standards in life-sciences ML, highlighting the need for evaluation designs aligned with the biological sampling structure [13,14,15].

In this study, we address these limitations with a reproducibility-oriented framework that integrates expression-related and mutation-derived features from scRNA-seq data. The revised analysis emphasizes a GATK-centered gene-burden workflow, leakage-safe validation, exact GSM-level permutation testing, and split-identical comparison of variant-only, expression-only, and combined feature sets. Run-level CV is reported as a separability comparator, whereas GSM-grouped validation is used as the primary independence-aware check.

The main contributions of this work are as follows:

1. A GATK-centered, auditable workflow for deriving RNA-seq mutation-derived gene-burden features from scRNA-seq data;
2. A split-first validation design comparing run-level CV with GSM-grouped leave-one-GSM-out validation and exact GSM-level permutation testing;
3. A feature-set comparison of GATK variant-only, expression-only, and combined feature matrices under identical grouped splits;
4. A cautious expression–mutation interpretation framework, including FDR-aware marker-overlap reporting and external-dataset scope definitions.

## 2. Results

### 2.1. Dataset Composition and Cohort Summary

Table 1 summarizes the run-level class distribution. The dataset comprises 14 sequencing runs from seven donor proxies: eight runs were assigned to unwounded skin and six to the wound edge. The two-runs-per-donor structure encodes both biological (condition-level) and technical (within-donor run-pair) variation and motivates the explicit split sensitivity analysis described below.

Donor-level metadata revealed an age range of 23–69 years (median 42.0 years), balanced sex composition (4F/3M), and a two-run structure per donor that introduces within-donor technical correlation. This structure is a key driver of the performance gap between run-level and donor-level evaluation discussed in Section 3.4.

The paired run structure per donor introduces inherent dependencies between samples, which motivates the need for split-aware validation strategies considered in subsequent analyses.

Supplementary Table S1 maps every SRR run to its GSM/donor proxy, condition, available age/sex metadata, and leave-one-GSM-out fold assignment. The table shows that each GSM/donor proxy contributes two SRR runs and that both runs are held out together in the same grouped-validation fold.

### 2.2. Variant Count Distribution and Cohort Filtering

Table 2 presents the GATK primary variant-record summary. Across 14 runs, the GATK PASS branch yielded a median of 1,272,246.5 records per sample (range 608,759–1,549,617), with median SNP and indel counts of 1,123,419 and 133,470, respectively. After cohort-common filtering, the median decreased to 387,333.5 records (range 210,199–474,211), corresponding to a median per-sample reduction of 69.555%. Median SNP and indel reductions were 67.942% and 81.111%, respectively.

We removed the prior condition-stratified interpretation of raw variant-count differences because RNA-seq variant counts are strongly affected by coverage, expression level, mapping artifacts, and filtering choices. In the revised results, variant-count summaries are used for workflow/QC reporting and feature-space documentation, not as evidence of condition-specific mutation burden or biomarker status.

Wide inter-sample dispersion persisted after GATK cohort filtering: the cohort-filtered branch ranged from 210,199 to 474,211 records per sample (2.26× range). This confirms that cohort-frequency filtering substantially reduces the shared/background variant space while preserving a large mutation-derived feature space for downstream gene-burden modeling. Exact per-sample values are retained in the study audit outputs.

**Figure 1:**
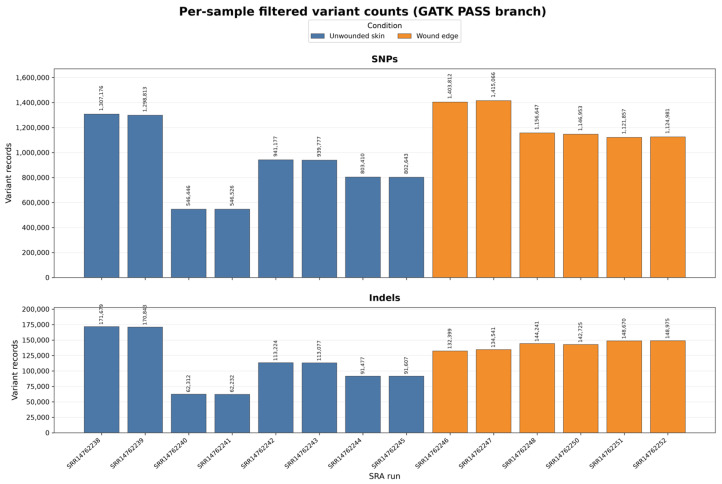
Per-sample filtered variant counts (GATK-filtered branch). Y-axis: total PASS variant records; X-axis: SRA run identifiers. Colors distinguish condition labels (wound edge vs. unwounded skin).

Overall, cohort-frequency filtering is now described as a dimensionality and background-signal reduction step. We avoid claiming that it proves improved biological signal; its practical impact is evaluated downstream through run-level and GSM-grouped validation. The per-sample GATK-filtered variant counts are shown in Figure 1.

**Figure 2:**
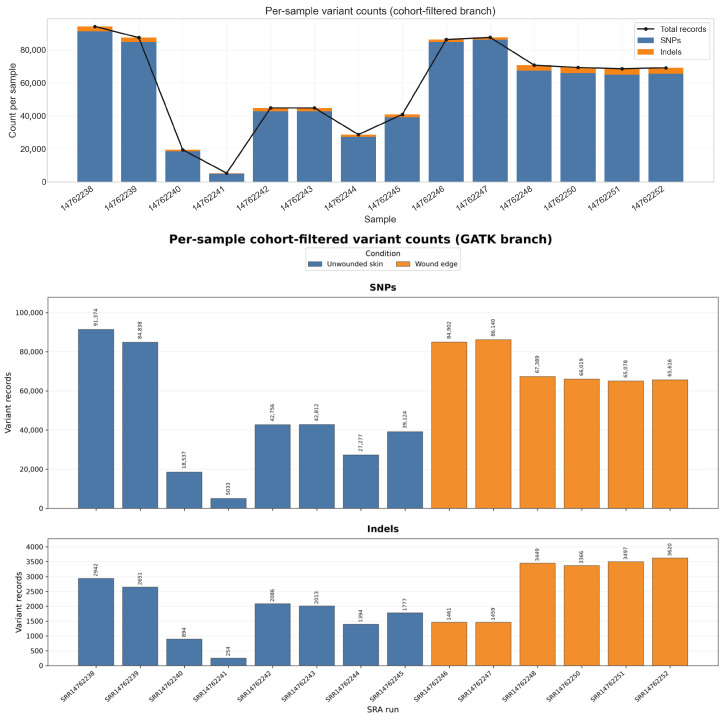
Per-sample cohort-filtered variant counts. Note the proportional downward shift relative to Figure 1, while inter-sample rank ordering is largely preserved, illustrating that cohort-frequency filtering reduces the shared/background variant space without wholesale reordering of the burden landscape.

After cohort-common filtering, the corresponding per-sample counts decreased proportionally (Figure 2).

### 2.3. Workflow-Level Sensitivity: BCFtools vs. GATK

Table 3 presents a workflow-level sensitivity comparison between the archived BCFtools baseline and the active GATK primary workflow. GATK PASS records totaled 16,283,367 compared with 1,698,411 archived BCFtools filtered records (9.587×). After cohort filtering, the GATK per-sample total was 4,964,502 compared with 817,043 for BCFtools (6.076×). GATK also produced more unique cohort-common loci (882,064 vs. 141,773; 6.222×) and more genes with non-zero burden (27,230 vs. 18,860; 1.444×). Because preprocessing, filtering, and caller behavior differ between branches, this comparison is interpreted as workflow-level sensitivity rather than a pure caller-only benchmark.

These differences underscore that variant callers, preprocessing, and filtering choices are first-class methodological decisions in scRNA-seq mutation-based ML. The stable conclusion is not that one branch provides a validated truth set, but that mutation-derived gene-burden features are workflow-dependent; therefore, classification and validation claims in the manuscript are tied to the GATK primary branch, with BCFtools retained only as a sensitivity context.

### 2.4. Run-Level Separability and GSM-Grouped Validation

From a machine learning perspective, the run-level cross-validation results indicate apparent within-dataset separability between conditions; however, this estimate is potentially optimistic until GSM-grouped validation is considered.

Table 4 summarizes the revised GATK validation results. The GATK gene-burden matrix contained 27,230 features across 14 runs. Run-level repeated stratified cross-validation produced high within-dataset separability (balanced accuracy 0.973 +/− 0.113), but this result is treated as a comparator rather than evidence of independent generalization. GSM-grouped leave-one-GSM-out validation produced a balanced accuracy of 0.708, and exact GSM-grouped permutation testing was not significant (*p* = 0.257).

The revised permutation analysis is interpreted at the GSM group level. Exact class-balanced grouped permutation testing produced *p* = 0.257 for balanced accuracy and *p* = 0.286 for ROC AUC. Thus, although the run-level model shows high within-dataset separability, the grouped permutation result does not support a strong independent classifier-performance claim in this small cohort.

Table 5 reports the revised logistic-regression validation summary for the GATK gene-burden matrix. The run-level model achieved a balanced accuracy of 0.973 and an ROC AUC of 0.980, while GSM-grouped validation achieved a balanced accuracy of 0.708 and an ROC AUC of 0.750. Exact grouped permutation testing gave *p* = 0.257 for balanced accuracy and *p* = 0.286 for ROC AUC, so grouped validation is interpreted cautiously in this small cohort.

**Figure 3:**
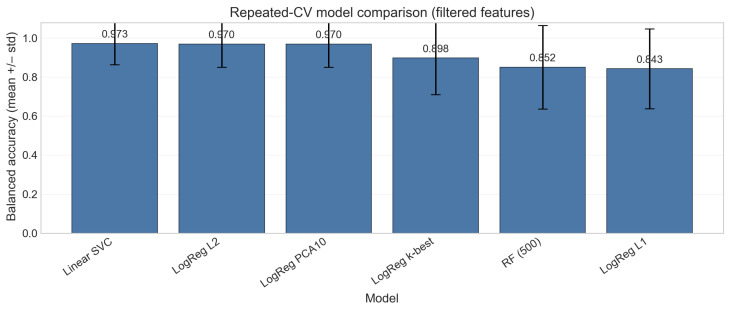
Run-level repeated-CV balanced accuracy distribution for the GATK gene-burden matrix. This figure should be interpreted as within-dataset separability, not independent group-level generalization.

**Figure 4:**
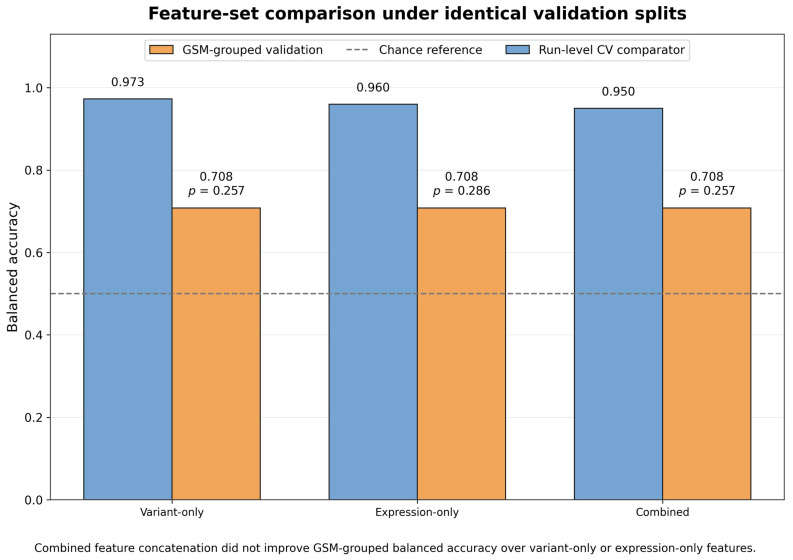
Feature-set comparison under identical validation splits. Run-level CV remains high for all feature sets, whereas GSM-grouped balanced accuracy is 0.708 for variant-only, expression-only, and combined features; exact grouped permutation *p*-values are shown above grouped bars. The identical grouped balanced accuracy reflects identical hard GSM-level decisions across feature sets (5/7 GSM groups correct, with Sc_Skin1 false-positive and Sc_W2 false-negative), while probability ranking differed across feature sets as reflected by ROC-AUC.

Figure 3 is retained only as a run-level separability diagnostic. Figure 4 now summarizes the final feature-set comparison under identical split definitions, showing that simple concatenation of variant-derived and expression features does not improve GSM-grouped balanced accuracy in this small cohort.

These results suggest that the model captures non-random structure in the data; however, this performance must be interpreted in the context of potential data dependencies, as explored in the following section.

### 2.5. Split-Sensitivity and Leakage Analysis

To evaluate performance under an independence-aware grouped design, split sensitivity was analyzed by comparing run-level and GSM-grouped evaluation strategies.

Current GATK validation shows run-level repeated stratified CV balanced accuracy = 0.973 +/− 0.113, while GSM-grouped leave-one-GSM-out validation gives balanced accuracy = 0.708. The mean same-GSM train/test overlap in run-level CV was 0.884, whereas GSM-grouped validation removes this overlap. The numerical comparison of the two evaluation protocols is summarized in Table 6.

The current GSM-grouped result is above chance numerically (balanced accuracy 0.708), but the exact grouped permutation is not significant (*p* = 0.257). Two GSM groups are misclassified (Sc_Skin1 and Sc_W2).

To make the grouped validation transparent at the independent GSM/donor-proxy level, Supplementary Table S3 reports each leave-one-GSM-out fold, held-out GSM/donor proxy, true condition, predicted condition, mean wound probability, mean decision score, and fold-level result.

Supplementary Table S4 reports the exact GSM-aggregated confusion matrix and a small-cohort uncertainty summary. Group-level accuracy is 5/7 (0.714), with a wide Wilson 95% CI of 0.359–0.918; GSM-grouped balanced accuracy is 0.708 and exact grouped permutation is not significant (*p* = 0.257).

An expression-only baseline was added using the processed pseudobulk gene-expression matrix transformed as sample-wise log1p(CPM). The same leakage-safe validation design was used: run-level repeated stratified CV as a separability comparator, GSM-grouped leave-one-GSM-out validation as the independence-aware check, and exact GSM-level permutation testing. Expression-only features showed high run-level separability (balanced accuracy 0.960 +/− 0.133; ROC-AUC 1.000 +/− 0.000) but weaker GSM-grouped performance (balanced accuracy 0.708; ROC-AUC 0.500; exact grouped permutation *p* = 0.286 for balanced accuracy). Because automatic cell-identification methods can vary in performance across scRNA-seq datasets and algorithms [16], cell-type annotations were used only as contextual expression information and not as the primary classification endpoint.

We then compared GATK variant-derived gene-burden features, expression-only features, and a simple combined GATK-variant-plus-expression feature set under identical leakage-safe splits. The combined matrix used prefixed feature identifiers and concatenated the GATK gene-burden matrix with the log1p(CPM) expression matrix. Under the primary GSM-grouped analysis, variant-only and combined features produced the same balanced accuracy (0.708), ROC-AUC (0.750), and exact grouped permutation *p*-value for balanced accuracy (*p* = 0.257). Expression-only features also had a balanced accuracy of 0.708 but an ROC-AUC of 0.500 and *p* = 0.286. The repeated balanced-accuracy value is expected from the seven-group validation design because all three feature sets made the same two hard GSM-level errors: Sc_Skin1 was predicted as a wound edge and Sc_W2 was predicted as unwounded skin. Thus, sensitivity was 2/3 and specificity was 3/4 for each feature set, giving balanced accuracy (2/3 + 3/4)/2 = 0.708, despite differences in prediction probabilities and ROC-AUC.

**Figure 5:**
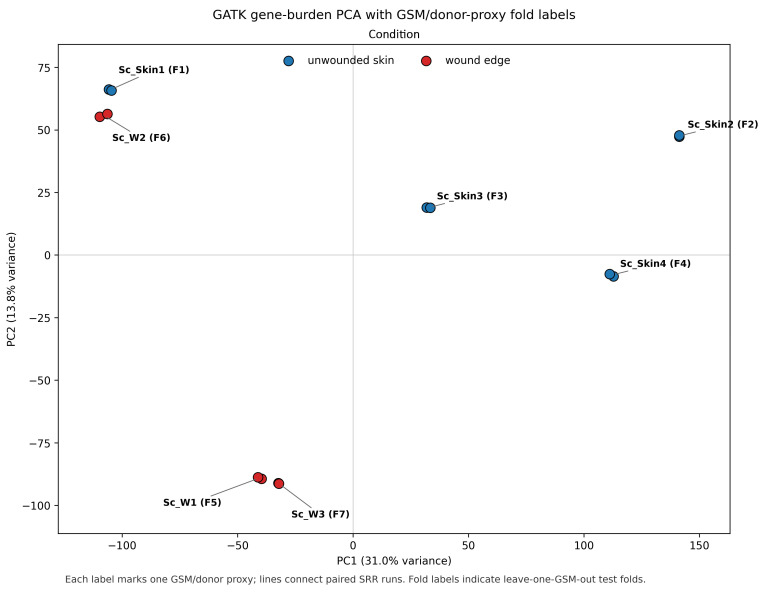
GATK gene-burden PCA with GSM/donor-proxy fold labels. Each label marks one GSM/donor proxy; lines connect paired SRR runs from the same GSM, and fold labels indicate the leave-one-GSM-out test folds.

**Figure 6:**
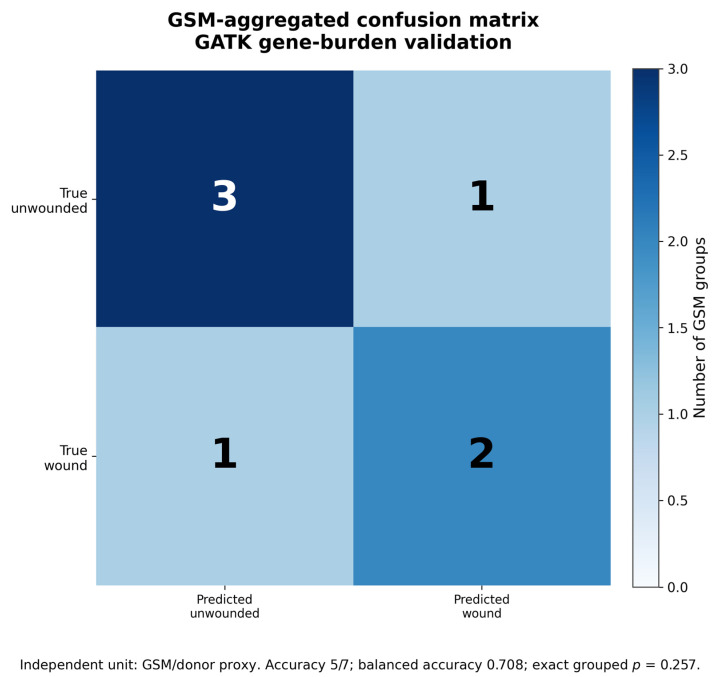
GSM-aggregated confusion matrix for the primary GATK gene-burden grouped validation. The independent unit is the GSM/donor proxy; group-level accuracy is 5/7, balanced accuracy is 0.708, and exact grouped permutation *p* = 0.257.

The updated visualization now focuses on the final GATK analysis branch. Figure 5 shows the GATK gene-burden PCA with paired SRR runs connected and each GSM/donor-proxy fold labeled. Figure 6 reports the GSM-aggregated confusion matrix for the primary grouped validation.

The mechanistic interpretation of this finding is straightforward: the gene-burden feature vectors encode substantial within-donor technical consistency (shared sequencing batch, library prep, and alignment conditions for paired runs), which naive CV exploits as a discriminative signal but which is absent in a true out-of-donor test scenario. Without an explicit donor-aware split design, this structure produces an optimistic performance illusion.

This substantial performance gap indicates that run-level validation captures donor-specific patterns rather than true biological differences, leading to overestimation of model performance.

### 2.6. Expression–Mutation Correlation Analysis

Table 7 presents the Spearman correlation matrix among three run-level burden metrics. Total gene burden was positively associated with both filtered records (ρ = 0.684) and cohort-filtered records (ρ = 0.837). The strongest pairwise association was between filtered and cohort-filtered record counts (ρ = 0.947), confirming that cohort filtering preserves the relative burden structure across samples rather than introducing substantial reordering. These correlations indicate that samples with high total variant burden tend to retain proportionally more records after cohort filtering, suggesting that within-donor technical variation, rather than systematic cohort-common locus removal, drives count variability.

Hypergeometric marker-overlap enrichment testing comprised 114 tests (38 clusters × 3 mutation-derived gene sets). Of these, only 13 tests yielded any overlap (overlap_n > 0), and only 1 test reached nominal significance (*p* < 0.05): cluster 8 (T cell) against wound_edge_higher_mutation_burden, with the single overlap gene *IL7R* (*p* = 0.042, q = 0.631). No test survived FDR correction (q < 0.05: 0 tests).

The sparsity and non-significance of marker-mutation overlaps are consistent with the expectation that somatically expressed variants detected through scRNA-seq variant calling are not systematically co-localized with transcriptional cluster markers. Expressed variants capture a different biological layer (somatic mosaicism, RNA editing, alignment artifacts, or low-frequency germline variation) than the differentially expressed genes that define transcriptional clusters. The absence of significant overlap after FDR correction should not be interpreted as a negative biological finding but rather as confirmation that these two feature types are largely orthogonal.

### 2.7. Functional Enrichment Context

Table 8 summarizes top enrichment terms for three mutation-derived gene sets, included as contextual annotation rather than causal biological claims. The top_mutated_genes set was enriched for broad cellular compartment terms (cytoplasm, cytosol) and general molecular function terms (ion binding, small molecule binding), reflecting the broad genomic distribution of expressed variants rather than a specific biological program.

The wound_edge_higher_mutation_burden set showed enrichment for immune-associated categories, including MHC protein complex (GO:0042611; *p* = 7.69 × 10^−8^), allograft rejection (KEGG:05330; *p* = 2.35 × 10^−6^), and antigen binding (GO:0003823; *p* = 4.02 × 10^−7^). These terms are biologically plausible in the wound context, where immune activation and antigen presentation are expected, but they do not constitute evidence of direct mechanistic involvement of the variant-harboring genes.

**Figure 7:**
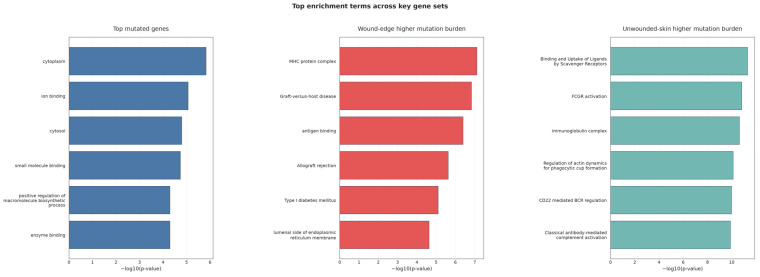
Summary of top enrichment terms by mutation-derived gene set. This figure should be interpreted as contextual annotation; enrichment *p*-values reflect the overall mutated gene-set composition and are not evidence of condition-specific functional activation.

The unwounded_skin_higher_mutation_burden set showed enrichment for scavenger receptor binding (REAC:R-HSA-2173782; *p* = 4.90 × 10^−12^), FCGR activation (REAC:R-HSA-2029481; *p* = 1.49 × 10^−11^), and immunoglobulin complex (GO:0019814; *p* = 2.29 × 10^−11^). The prominence of phagocytosis-related and Fc-receptor-associated pathways in unwounded skin may reflect baseline immune surveillance activity in resting skin tissue, as summarized in Figure 7.

These findings indicate that mutation-derived metrics retain structured run-level information, but this structure is interpreted as exploratory and must be evaluated with group-aware validation before being treated as predictive evidence.

Overall, the results show that standard run-level validation can substantially overstate apparent separability in a small-cohort scRNA-seq setting. The expression–mutation analyses provide exploratory system-level context, but the current data do not support a strong biomarker or independently validated classifier claim.

## 3. Discussion

### 3.1. Integration of Expression and Mutation Signals

The integration of heterogeneous biological signals remains a central challenge in single-cell analysis. While scRNA-seq studies traditionally focus on transcriptional profiles, recent work has highlighted the importance of incorporating additional modalities, including mutation-derived signals, to better characterize cellular heterogeneity [6,7]. At the same time, multi-omics integration frameworks such as MOFA+ and multimodal single-cell approaches demonstrate the value of combining complementary data sources for improved biological interpretation [8,9].

In this study, we proposed a unified framework that integrates gene expression–related information with mutation-derived features extracted from scRNA-seq data. Our results show that gene-level overlap between expression markers and mutation-derived gene sets is minimal after multiple testing correction, which is consistent with previous findings that transcriptional and genetic variation signals reflect distinct biological processes [6,7].

System-level correlations suggest that mutation-derived metrics and expression-derived summaries can provide complementary exploratory context. However, the split-identical feature-set comparison showed that simple concatenation of GATK gene-burden and expression features did not improve GSM-grouped balanced accuracy over either feature set alone. Therefore, complementarity is presented as a hypothesis-generating biological observation rather than as independently validated added predictive value.

### 3.2. Implications for Machine Learning in Biomedical Data

A major finding of this study is the discrepancy between run-level and GSM-grouped validation results. Conventional run-level cross-validation yielded high apparent separability, whereas GSM-grouped evaluation reduced performance relative to run-level cross-validation and was not statistically significant under exact grouped permutation testing.

This effect reflects a well-known challenge in biomedical machine learning related to data leakage and overestimation of model performance [12]. When samples are not independent—such as multiple runs originating from the same donor—standard validation strategies can introduce hidden dependencies between training and test sets, leading to overly optimistic results [11,17].

The observed performance gap highlights the importance of structured validation strategies in omics data analysis. Similar concerns have been raised in recent studies emphasizing reproducibility and validation design in machine learning for life sciences [13,15]. In this small-cohort dataset, model performance should be interpreted cautiously, and biologically informed validation protocols are essential.

### 3.3. Role of Feature Engineering and Data Integration

Gene-level aggregation of mutation-derived records provides a transparent dimensionality-reduction step and produces a feature matrix that can be audited across callers, filters, and validation designs. This representation is useful for method comparison, but biological specificity and classifier utility must be established empirically rather than assumed from aggregation alone. This distinction between statistical inference and predictive machine learning is important when interpreting small-sample biomedical analyses [18].

Cohort-frequency filtering is therefore described as a background/common-locus reduction step rather than proof of improved biological signal. The interpretation focuses on how workflow choices affect feature-space size, run-level separability, and GSM-grouped validation.

The practical value of integrating mutation-derived and expression-derived features in this cohort is methodological and exploratory: it enables split-identical comparisons across feature modalities, but larger independent cohorts are required before claims about predictive added value or transferable biological signatures can be made. Transparent and interpretable reporting is therefore necessary when integrating multiple feature modalities [19].

### 3.4. Limitations and Future Directions

Several limitations should be considered. First, the study includes only 14 sequencing runs from 7 GSM/donor proxies, which limits statistical power and makes grouped-validation estimates sensitive to individual donor-level units. Second, GSM identifiers are used as donor proxies, and donor-proxy uncertainty should be considered when interpreting group-aware validation. Third, variant calls were derived from RNA-seq data and may be affected by coverage, expression level, allele-specific expression, RNA editing, strand bias, mapping artifacts, and base-quality effects. Fourth, the variant calls were not orthogonally validated using DNA sequencing or targeted assays. Fifth, no independent raw external validation cohort is claimed for the GATK mutation-derived classifier. Sixth, expression–mutation marker overlap was not FDR-significant across the 114 hypergeometric tests, so biological convergence interpretations remain exploratory. Finally, GSM-grouped validation produced a balanced accuracy of 0.708, but exact grouped permutation was not significant (*p* = 0.257); therefore, classifier-performance claims should be interpreted cautiously.

To avoid overclaiming external validation, we screened public wound/ulcer single-cell datasets and classified their permissible use in the study. GSE248247 is treated only as raw 10× feasibility evidence because it contains two ulcer samples and does not provide a sufficiently powered independent validation cohort. GSE165816 and GSE265972 are treated as processed biological/expression context only because GEO does not provide open raw data suitable for direct reprocessing through the GATK mutation-derived pipeline.

Third, the current framework relies on classical machine learning models. Although deep learning approaches have shown strong potential in genomic data analysis [20,21,22], their application in small datasets remains challenging due to overfitting and interpretability issues.

Future work should focus on extending the framework to larger datasets and incorporating additional data modalities. Advanced integration strategies, including graph-based and trajectory-based approaches, may further improve the modeling of complex biological relationships [23].

### 3.5. Practical Implications

The results of this study have practical implications for machine-learning pipeline design in biomedical research. Specifically, they show that high performance under standard validation protocols may not reflect performance under independent group-aware validation.

The dependence of results on validation strategy highlights the need for GSM/donor-aware evaluation in small-cohort omics studies. Integration of heterogeneous biological signals remains useful for exploratory interpretation, but predictive conclusions should be separated from system-level biological context.

These findings are consistent with current recommendations for improving reproducibility and transparency in computational biology and machine learning research [13,15] and emphasize the importance of aligning analytical methods with the structure of biological data.

## 4. Materials and Methods

### 4.1. Dataset and Study Design

We used BioProject PRJNA736095 (NCBI SRA/GEO), which contains 14 scRNA-seq runs derived from 7 human donors with skin tissue sampled from two conditions: unwounded skin (*n* = 8 runs, 4 donor proxies) and wound edge (*n* = 6 runs, 3 donor proxies) [24]. Donor sex distribution was 4 females and 3 males, with an age range of 23–69 years (median 42.0 years). The two-run-per-donor design introduced an explicit within-donor technical replication structure that motivates donor-aware validation strategies.

The study followed an observational, retrospective design using publicly available, de-identified sequencing data. No patient recruitment, primary sample collection, or clinical intervention was performed. All analyses were conducted on derived computational outputs with full methodological transparency.

### 4.2. Read Alignment with STARsolo

Raw FASTQ reads were processed using STARsolo (version 2.7.11b) [25] for simultaneous alignment and cell barcode/UMI demultiplexing. STARsolo was selected for its compatibility with 10× Chromium library chemistry and its integrated single-cell preprocessing capacity. Genome indexing used GRCh38 (hg38) with matched GTF annotation; all alignment parameters were taken directly from project configuration files to ensure reproducibility.

Aligned reads were output in BAM format, sorted, and indexed with SAMtools (version 1.19.2) [26,27]. Duplicate marking and read group assignment followed GATK best-practice preprocessing recommendations for RNA-seq variant calling.

### 4.3. Variant Calling with GATK HaplotypeCaller

Variant calling used the GATK (version 4.6.2.0) RNA-seq variant-calling workflow [28,29], centered on per-sample HaplotypeCaller in GVCF mode, joint genotyping, and hard-filter PASS export. Filtering used GATK RNA-seq quality metrics including quality-by-depth, strand-bias, mapping-quality, and related criteria defined in the project configuration. Because RNA-derived variants can be affected by expression, coverage, mapping, editing, and allele-specific effects, the GATK branch is treated as the primary reproducible analysis workflow rather than an orthogonally validated DNA-variant truth set. General variant-calling quality-control principles, including attention to coverage, mapping quality, strand bias, and filtering, were considered when defining this workflow [30].

Active stage mode was import_existing, corresponding to re-analysis of pre-called GVCFs. The active GATK primary workflow was compared against an archived BCFtools (version 1.19, but 1.23 for SRR14762238) workflow as a workflow-level sensitivity analysis across variant records, gene coverage, and mutation burden (Table 3). Because preprocessing, filtering, and caller behavior may differ between branches, this comparison is not interpreted as pure caller-only concordance.

### 4.4. Cohort-Frequency Filtering

A secondary cohort-frequency filtering branch was constructed to examine how removing cohort-common loci changes feature-space size and downstream validation behavior. Cohort-common loci were defined as sites observed in at least 4 samples with maximum per-sample variant allele frequency (VAF) >= 0.05. These loci were excluded from the cohort-filtered branch. This filtering branch is used as a workflow-sensitivity and dimensionality-reduction check, not as proof that the retained variants are a condition-specific biological signal.

The cohort-frequency threshold was selected to target loci exhibiting near-ubiquitous presence in the study cohort, which are unlikely to be informative for condition-level discrimination and may introduce noise. The formal definition is given in Equation (1):

*L_common* = {*l* ∈ L | |{s ∈ S:VAF(l, s) ≥ 0.05}| ≥ 4} (1)

where L is the set of all loci, S is the set of samples, and VAF(l, s) denotes the variant allele frequency at locus l in sample s. The cohort-filtered feature set is constructed by removing all L_common loci from the input variant set.

### 4.5. Feature Engineering: Gene-Burden Vectors

The classification problem is defined as follows. Given a feature matrix X ∈ R^(*n* × *p*), where *n* is the number of samples and *p* is the number of features, and a label vector y ∈ {0,1} representing biological conditions, the task is to learn a function f: X → y.

Variants from each branch were mapped to genes using coordinate overlap against GRCh38 (release 43)GTF gene interval annotations [31]. For each sample, a gene-burden vector was constructed by summing the total number of variant records overlapping each gene. This produces a *p*-dimensional feature matrix X ∈ R^(*n* × *p*), where *n* = 14 samples and *p* equals the number of genes with a non-zero burden in at least one sample. Feature selection and dimensionality reduction are particularly important in high-dimensional scRNA-seq analysis [32].

The burden aggregation is defined in Equation (2):

b (s, g) = |{v ∈ V(s):chr(v) = chr(g), start(g) ≤ pos(v) ≤ end(g)}|(2)

where V(s) is the set of PASS variants in sample s, and b (s, g) is the total variant count mapped to gene g in sample s. This representation captures both the presence and relative density of somatic-like expressed variants at the gene level without requiring allele-specific phasing.

Feature engineering was performed by constructing gene-level representations from mutation-derived data and integrating them with expression-derived features, enabling a joint representation of biological signals.

### 4.6. Machine Learning Framework

Primary GATK validation was performed with a leakage-safe scikit-learn (version 1.4.1.post1) [33] pipeline composed of VarianceThreshold feature filtering, StandardScaler normalization, and L2-regularized logistic regression with class_weight = ‘balanced’. This revised primary model was selected because it can be fit consistently inside each validation fold in the high-dimensional, small-n setting. This revised primary model was selected because it can be fit consistently inside each validation fold in the high-dimensional, small-n setting, where feature-selection strategy can materially affect model behavior [34]. Earlier exploratory model-family comparisons, including linear SVC, random forest, PCA-based variants, and SelectKBest variants, are not used as the basis for the revised independence-aware conclusions.

Two validation protocols were reported. First, run-level repeated stratified cross-validation (5 folds × 30 repeats) was used as a within-dataset separability comparator. Second, GSM-grouped leave-one-group-out validation was used as the independence-aware analysis, with both runs from the same GSM/donor proxy kept together in either training or testing. Balanced accuracy and ROC AUC were reported for both protocols, but grouped validation is used for cautious interpretation of model performance.

For the expression-only baseline, the processed pseudobulk gene-expression matrix was normalized by sample-wise counts per million and transformed as log1p(CPM). The same leakage-safe validation function, GSM grouping, exact group-level permutation design, and fold-fitted pipeline were then applied to expression-only features.

For the combined feature-set comparator, GATK variant-derived and expression-derived feature rows were concatenated after adding source-specific prefixes to feature identifiers. The same GSM grouping, fold-fitted preprocessing/model pipeline, and exact GSM-level permutation procedure were used for the variant-only, expression-only, and combined matrices.

Statistical significance for the revised primary validation was assessed using exact GSM-grouped permutation testing. Permutation testing provides a direct non-parametric assessment of classifier performance under label exchangeability [35]. Class-balanced labels were permuted at the GSM/donor-proxy level (35 exact labelings), preserving the two-run group structure within each permutation. Run-level permutation testing is retained only as a within-dataset separability check and is not used as evidence of independent generalization. The grouped permutation *p*-value is defined in Equation (3):

Leakage-control rule. For every validation fold, train/test or group assignments were generated before any feature filtering, scaling, or model fitting. Within each training fold, VarianceThreshold, StandardScaler, and logistic-regression parameters were learned only from training samples and then applied to the held-out samples. No scaling, PCA, SelectKBest feature selection, model fitting, or model-selection step was fitted on the full dataset before validation. The same split-first rule was used for run-level CV, GSM-grouped validation, GSM-wise predictions, and grouped permutation testing.

The split-first validation rule is made explicit in Supplementary Table S2 and in the repository pseudocode output. For every fold, train/test or GSM-group assignments are created before preprocessing; VarianceThreshold, StandardScaler, and logistic-regression fitting are learned only from the training fold and then applied to held-out samples.

p_exact = |{pi in Pi_exact:score(pi) >= score_obs}|/|Pi_exact(3)

where Pi_exact is the set of all class-balanced GSM/donor-proxy labelings and the observed labeling is included.

In the revised exact GSM-grouped permutation test, score_obs is the observed grouped metric and score(pi) is the metric after assigning the wound label to one of the 35 possible sets of three GSM/donor-proxy groups among seven groups. The exact *p*-value is the fraction of class-balanced group labelings with score(pi) >= score_obs; for balanced accuracy this was 9/35 = 0.257, and for ROC AUC this was 10/35 = 0.286.

Supplementary Table S5 reports the exact GSM-grouped permutation tail counts used for grouped validation.

### 4.7. Split-Sensitivity Analysis

To explicitly quantify the gap between run-level and group-aware performance estimates, GSM-grouped leave-one-group-out evaluation was performed. GSM/donor-proxy IDs from external SRA/GEO metadata were used to define grouped evaluation, ensuring that both runs from the same GSM/donor proxy always appear in the same fold (train or test, never split across). This removes within-GSM run sharing between train and test sets and provides a more conservative group-aware performance estimate; it should not be interpreted as an external out-of-distribution benchmark.

The run-level vs. donor-level performance gap (Δ balanced accuracy) is the primary leakage-sensitivity metric reported in this study [17].

### 4.8. Expression–Mutation Correlation Module

This module represents the core methodological contribution of the study, enabling the integration of gene expression and mutation-derived features within a unified analytical framework.

1. Spearman’s rank correlation [36] was computed among three run-level metrics: total gene burden, filtered variant record count, and cohort-filtered record count (Table 7). Spearman correlation was selected for robustness to non-normality and outlier influence in small samples.
2. Marker-overlap hypergeometric enrichment was computed between expression-derived cluster marker sets (expression_top_markers_by_cluster.csv; 38 clusters) and three mutation-derived gene sets: top_mutated_genes, wound_edge_higher_mutation_burden, and unwounded_skin_higher_mutation_burden. Enrichment *p*-values were computed using the hypergeometric test and adjusted for multiple comparisons using the Benjamini–Hochberg FDR procedure [37]. The BH procedure controls the expected proportion of false positives among discoveries under independence and has become the standard approach for large-scale genomic testing [38]. The total test space was 38 clusters × 3 gene sets = 114 tests.

Functional annotation was performed using Profiler [39] against GO Biological Process, GO Molecular Function, GO Cellular Component, KEGG, and Reactome databases, with the fully expressed gene set as background. Results are reported as contextual sanity checks, not mechanistic proofs.

### 4.9. Workflow-Level Sensitivity Analysis of Variant-Calling Branches

Archived BCFtools workflow outputs were compared against the active GATK primary workflow across six cohort-level metrics: filtered/PASS records, filtered record range, cohort-common loci, variant-gene rows, genes with non-zero burden, and mutation-burden totals. This comparison contextualizes the quantitative consequences of workflow choice on downstream feature engineering and ML inputs. Because the branches may differ in preprocessing, references/annotations, filtering levels, and caller behavior, the analysis is reported as workflow-level sensitivity rather than a pure caller-only benchmark. Prior benchmarks have established substantial disagreement between BCFtools/mpileup and GATK HaplotypeCaller, particularly in sensitivity-specificity tradeoffs and false-positive rates [40,41].

The caller-by-caller methods/QC table documents the GATK primary workflow and archived BCFtools comparator separately, including reference/annotation, parameters, filtering, RNA-specific limitations, QC metrics, and conclusions that are stable versus workflow-dependent.

The caller/workflow comparison is reported as an explanatory sensitivity analysis. The GATK branch is the primary branch for validation and machine-learning interpretation, while the archived BCFtools branch is retained to document the workflow dependence of mutation-derived feature construction.

The overall workflow can be summarized as follows:

data acquisition → preprocessing → feature engineering → integration → classification → validation.

## 5. Conclusions

This study shows that GATK-derived gene-burden features from PRJNA736095 produce high apparent run-level separability but weaker, non-significant GSM-grouped validation. Specifically, run-level repeated stratified CV reached a balanced accuracy of 0.973 +/− 0.113, whereas GSM-grouped leave-one-GSM-out validation reached a balanced accuracy of 0.708 with exact grouped permutation *p* = 0.257. The central conclusion is therefore not that a robust classifier has been validated, but that standard run-level CV can substantially overstate apparent performance when repeated runs from the same GSM/donor proxy are split across train and test folds.

Expression-only and combined feature-set analyses under identical splits did not improve GSM-grouped balanced accuracy beyond 0.708, and expression–mutation marker overlap was not FDR-significant. Mutation-derived and expression-derived integration is therefore framed as exploratory and hypothesis-generating. Larger cohorts, independent raw external validation, and orthogonal DNA or targeted validation of RNA-derived variants are required before classifier-performance or biomarker claims can be made.

The pipeline, parameterization, analysis tables, and figures are provided to support auditability and reuse. The workflow can be adapted to other scRNA-seq classification problems where repeated runs or donor-related observations create implicit validation challenges, but transferability must be tested in larger independent cohorts.

**Figure 8:**
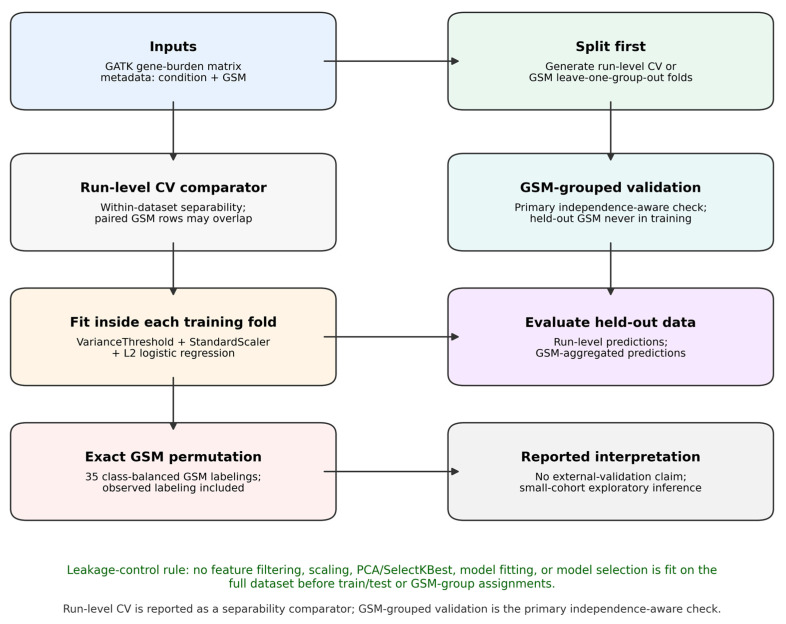
Leakage-safe validation workflow for expression processing, GATK mutation-derived gene-burden construction, and grouped model evaluation. Train/test or GSM-group assignments are generated before fold-dependent feature filtering, scaling, PCA/SelectKBest, model fitting, model selection, and permutation testing.

Pipeline Overview: The complete RNA-seq variant-calling pipeline is shown in Figure 8, highlighting preprocessing, variant calling using GATK HaplotypeCaller, and subsequent filtering steps.

## Figures and Tables

**Table 1:** Run-level sample counts by condition.

Condition	*n* (Runs)
Unwounded skin	8
Wound edge	6
Total	14

**Table 2:** Variant record summary by feature branch.

Feature Branch	*n*	Median Records	Min Records	Max Records	Median SNPs	Median Indels
Filtered (GATK PASS)	14	1,272,246.5	608,759	1,549,617	1,123,419	133,470
Cohort-filtered (GATK common)	14	387,333.5	210,199	474,211	360,150.5	25,210.5
Reduction from PASS to cohort	14	69.555%	-	-	67.942%	81.111%

**Table 3:** Workflow-level sensitivity comparison: BCFtools archived baseline vs. GATK primary workflow.

Metric	BCFtools (Archived Workflow)	GATK (Primary Workflow)	Δ (Abs)	Ratio	Direction
Filtered/PASS records, total	1,698,411	16,283,367	+14,584,956	9.587×	GATK primary branch retained more PASS records
Cohort-filtered per-sample records, total	817,043	4,964,502	+4,147,459	6.076×	GATK common-variant branch remains larger
Unique cohort-common loci	141,773	882,064	+740,291	6.222×	Caller sensitivity/filtering divergence
Genes with nonzero burden	18,860	27,230	+8370	1.444×	GATK matrix used for primary validation

**Table 4:** GATK validation summary: run-level separability comparator versus GSM-grouped validation.

Feature Branch	*n*	Features	Best Bal. Acc.	Permutation *p*	Best CV Model	Repeats	Perms.
GATK run-level repeated stratified CV	14	27,230	0.973 +/− 0.113	not primary	L2 logistic regression	30	-
GATK GSM-grouped leave-one-GSM-out	14	27,230	0.708	0.257	L2 logistic regression	7 groups	35 exact

**Table 5:** Revised GATK model-validation summary for run-level and GSM-grouped analyses.

Model	Bal. Acc. Mean	Bal. Acc. Std	ROC AUC Mean	Branch
Logistic regression (run-level CV)	0.973	0.113	0.980	GATK run-level separability comparator
Logistic regression (GSM-grouped LOGO, run-pooled)	0.708	-	0.750	GATK grouped validation
Logistic regression (GSM-grouped LOGO, GSM-aggregated)	0.708	-	0.750	GATK grouped validation
Exact GSM-grouped permutation	obs 0.708; *p* = 0.257	-	obs 0.750; *p* = 0.286	GATK grouped null test

Bal. Acc., balanced accuracy; Std, standard deviation; ROC AUC, area under the receiver operating characteristic curve; LOGO, leave-one-group-out.

**Table 6:** Split-sensitivity comparison: run-level vs. donor-grouped evaluation.

Evaluation Protocol	*n* Runs	*n* GSM Groups	Accuracy Mean	Bal. Acc. Mean
Run-level repeated stratified CV (5-fold × 30)	14	7	0.964	0.973
GSM-grouped leave-one-GSM-out	14	7	0.714	0.708
Absolute drop after GSM grouping	-	-	0.250	0.265 (27.2%)

Bal. Acc., balanced accuracy; CV, cross-validation; GSM, Gene Expression Omnibus sample; *n*, number of runs/groups.

**Table 7:** Spearman correlation matrix (run-level mutational burden metrics).

Metric	Total Gene Burden	Filtered Records	Cohort-Filt. Records
Total gene burden	1.000	0.684	0.837
Filtered records	0.684	1.000	0.947
Cohort-filtered records	0.837	0.947	1.000

Cohort-Filt. Records, cohort-filtered records.

**Table 8:** Top enrichment terms by mutation-derived gene set (g:profiler).

Gene Set	Term	Source	ID	*p*-Value
top_mutated_genes	Cytoplasm	GO:CC	GO:0005737	1.47 × 10^−6^
top_mutated_genes	Ion binding	GO:MF	GO:0043167	8.53 × 10^−6^
wound_edge_higher	MHC protein complex	GO:CC	GO:0042611	7.69 × 10^−8^
wound_edge_higher	Allograft rejection	KEGG	KEGG:05330	2.35 × 10^−6^
unwounded_skin_higher	Scavenger receptor binding	REAC	REAC:R-HSA-2173782	4.90 × 10^−12^
unwounded_skin_higher	FCGR activation	REAC	REAC:R-HSA-2029481	1.49 × 10^−11^

## Data Availability

The original sequencing data are openly available through NCBI BioProject PRJNA736095 (https://www.ncbi.nlm.nih.gov/bioproject/PRJNA736095; accessed 9 June 2026). Derived tables, figures, and analysis code are available in the project repository at https://github.com/dauletulyaidyn/somatic-like-scrna-variants-ml-pipeline (accessed 9 June 2026). Public datasets GSE248247, GSE165816, and GSE265972 were reviewed only for external-data scope and wording, as summarized in Supplementary Table S8.

## Supplementary Materials

The following supporting information can be downloaded at: https://www.mdpi.com/article/10.3390/ijms27146429/s1. Code and analysis workflows are available at https://github.com/dauletulyaidyn/somatic-like-scrna-variants-ml-pipeline (accessed on 9 June 2026).
